# Ecological Imprint of Rare Earth Mining on Microbial Communities and Water Quality Across Depth and Distance Gradients in Ganzhou, China

**DOI:** 10.3390/microorganisms13102236

**Published:** 2025-09-24

**Authors:** Yian Wang, Fei Shi, Fengxiang Lang, Guohua Wang, Yan Mao, Yingjie Xiao, Li Yin, Genhe He, Yonghui Liao

**Affiliations:** 1 Key Laboratory of Jiangxi Province for Functional Biology and Pollution Control in Red Soil Regions, School of Life Sciences, Jinggangshan University, Ji’an 343000, China; nickowya@163.com (Y.W.);; 2 Hydrology and Water Resources Monitoring Center of the Middle Reaches of Ganjiang River, Ji’an 343000, China

**Keywords:** rare earth elements, nitrogen, water quality, microbial communities, gene

## Abstract

Rare earth element (REE) mining exerts profound impacts on aquatic ecosystems, yet the microbial community responses and water quality under such stress remain underexplored. In this study, the surface (0.2 m) and subsurface (1.0 m) water along a spatial transect from proximal to distal points was investigated in a REE-mining area of Ganzhou, China. Physicochemical analyses revealed pronounced gradients of nitrogen (e.g., NH_4_^+^−N, NO_3_^−^−N), heavy metals (e.g., Mn, Zn, Pb), and REEs (e.g., La, Nd, Ce), with higher accumulation near mining sources and partial attenuation downstream. Dissolved oxygen and redox potential indicated mildly reducing conditions at contaminated points, potentially promoting denitrification and altering nitrogen cycling. Metagenomic sequencing showed significant shifts in microbial community composition, with enrichment of metal- and nitrogen-tolerant taxa, and key denitrifiers (e.g., *Acidovorax*, *Bradyrhizobium*, *Rhodanobacter*), particularly at upstream polluted points. KEGG-based gene annotation highlighted dynamic nitrogen transformations mediated by multiple pathways, including nitrification, denitrification, dissimilatory nitrate reduction to ammonium, and nitrogen fixation. Notably, genes associated with nitrite and nitrate reduction (e.g., *nir*, *nar*, *nrf*) were enriched near mining sources, indicating enhanced nitrogen conversion potential, while downstream activation of nitrogen-fixing genes suggested partial ecosystem recovery. Meanwhile, some microbial such as *Variovorax* carried metal tolerant genes (e.g., *ars*, *chr*, *cnr*). These findings demonstrate that REE and heavy metal contamination restructure microbial networks, modulate nitrogen cycling, and create localized ecological stress gradients. This study provides a comprehensive assessment of mining-related water pollution, microbial responses, and ecological risks, offering valuable insights for monitoring, restoration, and sustainable management of REE-impacted aquatic environments.

## 1. Introduction

Rare earth elements (REEs), encompassing a group of 17 chemically similar metals, have become essential to modern technologies, including electric vehicles, wind turbines, smartphones, and advanced defense systems [1]. Their unique magnetic, luminescent, and catalytic properties have led to an exponential increase in global demand over the past few decades. China, and, in particular, the southern Jiangxi Province (e.g., the Ganzhou region), has emerged as the world’s dominant supplier [2]. Much of this REEs extraction occurs through in situ leaching techniques that involve large-scale application of ammonium sulfate to weathered crust elution-deposited (ion-adsorption type) REE ores [3]. While efficient in resource recovery, this method is associated with widespread environmental degradation. Leaching processes mobilize not only REEs, but also co-occurring heavy metals such as lanthanum, cerium, and neodymium [4], as well as excess nitrogen species, particularly ammonium and nitrate [5]. These substances easily enter surrounding water bodies, creating persistent geochemical perturbations that extend downstream. The ecological implications of such disturbances, especially their impacts on water quality in surface/subsurface water systems that provide essential ecosystem services, remain need more characterized. Unlike traditional mining legacies (e.g., acid mine drainage), REE mining leaves a more subtle but potentially long-lasting chemical footprint—marked by increased metal loads and nitrogen enrichment in aquatic environments [6].

Microbial communities in surface/subsurface water systems are highly responsive to environmental changes and serve as both regulators and indicators of ecosystem health [7]. As the primary mediators of biogeochemical cycling—including nitrogen transformation, organic matter decomposition, and metal redox reactions—microorganisms underpin the resilience of surface/subsurface water ecosystems to anthropogenic stress [6]. However, the responses of microbial communities at different depths in surface and subsurface water along rare earth mining areas to the presence of nitrogen and metals have not been sufficiently explored. Most previous studies on mining impacts have focused on sediments, soils, or groundwater, neglecting the dynamic and stratified nature of microbial communities in surface waters [6,7,8]. In particular, two underexamined dimensions deserve attention: spatial distance from the mining source and vertical depth within the sampling points along the route. Spatial gradients can reflect dilution, sedimentation, or transformation processes along the transport pathway of contaminants, while depth-related differences in physicochemical variables such as nitrogen, metals, and dissolved oxygen further shape microbial niches and activity [9]. Although both spatial distance from the mining source and vertical depth are critical factors shaping water chemistry and microbial communities, studies that integrate these two dimensions remain scarce. Investigations that simultaneously address horizontal (distance) and vertical (depth) heterogeneity in mining-impacted surface and subsurface water microbiomes are therefore particularly valuable. Moreover, interactions between chemical gradients (e.g., metal and nitrogen levels) and microbial community structure are rarely treated as coupled systems. This limits our understanding of how mining activities influence not only community composition but also ecological function and potential feedback to water quality.

To enrich these key points, we conducted a field investigation in the REE-mining-impacted region of southern Jiangxi, targeting representative downstream points along the surface/subsurface water of a mining zone. Five sampling points were selected along a spatial transect from proximal to distal sites relative to mining activities, capturing the contaminant exposure. At each point, surface (0.2 m) and subsurface (1.0 m) water samples were collected to evaluate vertical differentiation. We simultaneously analyzed a suite of physicochemical parameters—including ammonium, nitrate, pH, dissolved oxygen (DO), and metal concentrations—alongside microbial community composition based on metagenomic sequencing. By integrating high-resolution microbial profiling with detailed chemical characterization, we aim to elucidate the ecological imprint of rare earth mining on surface and subsurface water ecosystems. This study contributes to a deeper understanding of how industrial extraction activities reshape biotic and abiotic components of aquatic environments, with implications for ecosystem monitoring, restoration, and sustainable resource management.

## 2. Materials and Methods

### 2.1. Study Area and Sampling

The study was conducted in the ion-adsorption rare earth mining region of Dingnan County, Ganzhou City, Jiangxi Province, China. This area, situated in a typical red-soil hilly landscape, has a subtropical humid monsoon climate, with a temperature of 28~29 °C and precipitation of 160~180 mm in August 2024 (Dingnan County People’s Government Network) [10]. Groundwater resources are abundant and occur mainly as Quaternary pore water in unconsolidated sediments and bedrock fissure water [11].

**Figure 1:**
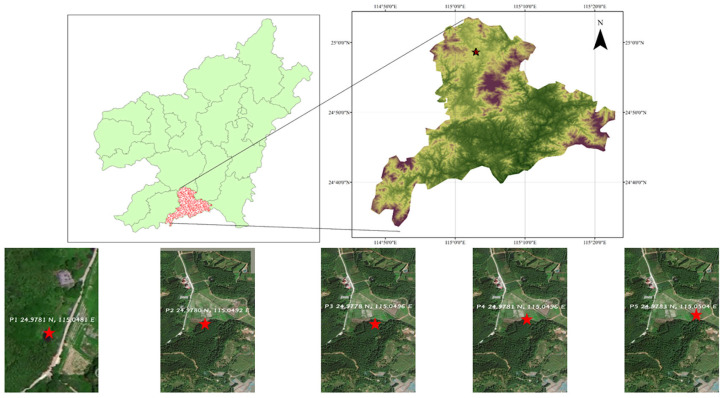
Rare earth mining area topographic map with five wells (P1–P5) in Dingnan County, Ganzhou City, Jiangxi Province, China.

Groundwater was sampled from five wells (depths of 2–13 m) located within (P2–P5, P2: 24.9780 N, 115.0492 E; P3: 24.9778 N, 115.0496 E; P4: 24.9781 N, 115.0496 E; P5: 24.9783 N, 115.0504 E) or outside (P1, CK: 24.9781 N, 115.0481 E) the mining area (Figure 1). At each point, 4 L of water was collected from 0.2 m and 1.0 m below the well water surface. All bottles were pre-rinsed three times with the corresponding sample water prior to collection. Samples for physicochemical analysis were collected in 500 mL polyethylene bottles and stored at 4 °C refrigerator in the laboratory until analysis.

### 2.2. Water Analysis Tests

Water samples collected from the rare earth mining area were filtered through 0.45 μm microporous fiber membranes immediately after collection. Each sample was partitioned for different analyses: one part was used to determine chemical oxygen demand (COD), total nitrogen (TN), ammonium nitrogen (NH_4_^+^−N), nitrate nitrogen (NO_3_^−^−N), fluoride (F^−^), and sulfate (SO_4_^2−^) contents; another part was acidified with high-purity nitric acid (0.5%, *v*/*v*) and stored in polyethylene bottles for trace element determination, including rare earth elements (REEs: La, Ce, Pr, Nd, Sm, Eu, Gd, Tb, Dy, Ho, Er, Tm, Yb, Lu, Sc, and Y) and metals (As, Fe, Mn, Cu, Zn, Pb, Cd, and Cr) contents. All samples were kept at 4 °C until analysis.

In situ measurements of dissolved oxygen (DO), pH, temperature, and oxidation-reduction potential (ORP) were conducted at each sampling point using a portable parallel analyzer (HACH SL1000, Hach Company, Loveland, CO, USA). COD content was determined by using the dichromate method (HJ 828−2017). TN and NH_4_^+^−N contents were quantified using a gas-phase molecular absorption spectrometer (GMA3212, Beiyu Technologies, Shanghai, China), while NO_3_^−^−N, F^−^, and SO_4_^2−^ contents were measured by ion chromatography (Metrohm Advanced IC-861, Herisau, Switzerland). Contents of Cu, Zn, Pb, Cd, Cr, and all REEs were determined by inductively coupled plasma–optical emission spectrometry (ICP-OES, Optima 8000, PerkinElmer, Waltham, MA, USA). As content was measured by using an atomic fluorescence spectrometer (AFS-9560, Haiguang Instrument, Beijing, China), and Fe, and Mn contents were measured by using an atomic absorption spectrophotometer (A3AFG-12, PERSEE, Beijing, China). Calibration was performed using serial dilutions of a high-purity ICP multi-element standard solution (100 mg/L, 5% HNO_3_; national standard material GSB04-1789-2004).

### 2.3. DNA Extraction and Metagenomic Analysis

For bacterial enrichment, each 2 L water sample was filtered through 0.22 μm mixed cellulose ester filters (Jingteng, Wuhan, China). Total genomic DNA was extracted from samples using the DNeasy PowerBiofilm Kit (QIAGEN, Hilden, Germany), and DNA quality and quantity were assessed with a NanoDrop One spectrophotometer. For library preparation, 0.2 μg DNA per sample was fragmented to ~350 bp using a Covaris LE220R-plus (Covaris, Woburn, MA, USA), end-polished, A-tailed, and ligated to Illumina adapters, followed by PCR amplification and purification with the AMPure XP system (Beckman Coulter, Brea, CA, USA) [12]. Library quality was evaluated with the Agilent 5400 system (Agilent Technologies, Inc., Santa Clara, CA, USA) and quantified by qPCR. Pooled libraries were sequenced on an Illumina platform (paired-end, 2 × 150 bp) at Novogene Bioinformatics Technology Co., Ltd. (Beijing, China), generating ~10 Gbp of raw data per sample. Predicted proteins were annotated against the Kyoto Encyclopedia of Genes and Genomes (KEGG) and NCBI-Nr databases using BLAST version 2.16.0. Metagenome-assembled genomes (MAGs) were taxonomically classified with GTDB-Tk v0.3.2 (release 89) [13], and gene abundance was expressed as transcripts per kilobase per million mapped reads (TPM). Meanwhile, species annotation was performed at the gene level to link functional potential. Taxonomic annotations were obtained using the LCA algorithm in MEGAN, with community composition summarized at the phylum and genus level. Chao1, Shannon, Simpson, and Ace diversity indices were calculated.

## 3. Results and Discussion

### 3.1. Spatial Patterns of Water Chemistry and Environmental Parameters

REE mining generates measurable gradients in water chemistry that persist over distance and depth. Physicochemical monitoring of water samples collected at 0.2 m and 1.0 m depths from five points along the mining area (P1–P5, P1 outside the mining area as CK) revealed significant impacts of mining effluents on water quality. TN contents (Table 1) exhibited pronounced spatial variation, with the reference site P1 showing 4.99–5.18 mg/L, whereas points near the mining area reached 88.2–90.7 mg/L at P2 and 102.6–103.4 mg/L at P3, indicating a substantial increase in nitrogen loading. Similar trends were observed for NH_4_^+^−N and NO_3_^−^−N (Table 1), with P3 NO_3_^−^−N reaching 104.1–107.7 mg/L, compared with 6.1–6.5 mg/L at P1, suggesting nitrate-dominated nitrogen pollution likely associated with tailings seepage, and direct wastewater discharge [14]. Pollutant contents decreased downstream, reflecting the effect of metabolic activity of soil microbial communities along the route.

COD levels near the mining points were markedly elevated, with P2–P3 at 1.0–1.9 mg/L and P4–P5 up to 1.6–4.2 mg/L, compared with 1.5–1.8 mg/L at P1 (Figure S1), indicating that downstream sections are additionally influenced by organic inputs from domestic, agricultural, or riverbank surface sources [15]. Nevertheless, the DO levels across P1–P3 (2.05–6.21 mg/L, Figure S2) remained within oxic rather than hypoxic ranges (DO ≈ 0.5 mg/L), which may explain the comparable TN and NO_3_^−^−N contents, as denitrification typically occurs under hypoxic or anaerobic conditions [16,17]. The higher ORP at P2–P3 (791.7–880.7 mV) than at P1 (429.0–452.7 mV) (Figure S3) may be attributed to their elevated Mn contents (2.127–3.393 mg/L; Mn^3+^/Mn^2+^ and MnO_2_/Mn^2+^ couples have a potential of +1.51 V and +1.23 V vs. SHE, respectively) compared with P1 (0.347–0.447 mg/L) and other metals across P1–P5 (0–0.647 mg/L). SO_4_^2−^ contents were higher at P1 than at P2–P5, likely reflecting natural geochemical background conditions. This may be attributed to agricultural activities near P1 (e.g., application of ammonium sulfate and potassium sulfate fertilizers) [18] and/or the occurrence of sulfur-bearing minerals such as pyrite (FeS_2_) [19] and gypsum (CaSO_4_·2H_2_O) [20] in the surrounding strata, whose weathering or dissolution can release abundant SO_4_^2−^ even in the absence of mining activities. Contents of SO_4_^2−^ (up to 124.7 mg/L) and F^−^ (up to 0.36 mg/L) were also elevated near the mining area (P2–P5) (Table 1 and Figure S4), reflecting increased inorganic ion loading. The elevated SO_4_^2−^ and NH_4_^+^−N contents near the mining area likely reflect the application of ammonium sulfate in REEs ore leaching, which introduces both SO_4_^2−^ and NH_4_^+^−N into surrounding waters, thereby influencing local water quality [21]. Moreover, F^−^ was included in the analyses because fluorine-bearing minerals (e.g., fluorite) are commonly associated with REE ores, and leaching could potentially release F^−^ into surrounding waters [22]. However, in our study, the F^−^ concentrations remained low (≤0.36 mg/L) with no substantial differences among sites, indicating minimal impact of mining activity on fluoride levels. Temperature (Figure S5) and pH (Figure S6) remained relatively stable, though local weakly acid pH appeared at P3 and P5, potentially influencing pollutant transport and biogeochemical processes [23]. Overall, mining activities produced localized gradients of high nitrogen, inorganic ion accumulation, and mildly reducing conditions, with slightly higher contamination at 1.0 m depth, suggesting downward penetration of pollutants.

### 3.2. Distribution Characteristics of Heavy Metals and Rare Earth Elements

Monitoring of heavy metals revealed significant contamination in mining-affected waters, particularly for Mn, Zn, Pb, and REEs. At the point P1, contents were near natural background levels (Mn 0.347–0.447 mg/L; Zn 0.058–0.063 mg/L; Pb 0.004–0.132 mg/L) (Table S1), indicating minimal influence. In contrast, near-source points P2 and P3 exhibited increased levels (Mn 2.127–3.393 mg/L; Zn 0.363–0.535 mg/L; Pb up to 0.195 mg/L). Downstream points P4 and P5 showed decreased metal contents, suggesting partial self-purification, although local accumulation existed. These results indicate that tailings leakage and wastewater discharge are primary sources of metals, which diffuse along the surface/subsurface water and may bioaccumulate in sediments or biota, posing ecological risks [24]. REE analysis further highlighted mining impacts. At P1, REEs were minimal (0.020–0.024 mg/L) than P2 and P3, and P2 and P3 showed REEs marked enrichment (e.g., La 0.2 m: 0.173–0.982 mg/L, 1.0 m: 0.208–1.063 mg/L; Nd 0.2 m: 0.082–0.644 mg/L, 1.0 m: 0.089–0.691 mg/L; Y 0.2 m: 0.088–0.537 mg/L, 1.0 m: 0.113–0.588 mg/L; Ce 0.2 m: 0.055–0.189 mg/L, 1.0 m: 0.059–0.201 mg/L) (Table S2), indicating REEs accumulation and a tendency for deeper layer enrichment. Downstream contents at P4–P5 were lower but remained above the reference point P1, reflecting gradual dilution along the flow path. These findings suggest that REEs, like conventional heavy metals, can serve as sensitive indicators of mining-related contamination and should be incorporated into environmental monitoring and water quality management frameworks [25].

In rare earth mining-impacted waters, the presence of REEs (La, Nd, Y, Ce) and heavy metals (Mn, Cu, Zn, Pb, Cd) may exerts the influence on nitrogen cycling by altering microbial activities and redox processes [26,27]. Excessive REEs exert cytotoxic effects, reducing the activity of nitrate and nitrite reductases and suppressing denitrification [28], consistent with the elevated NO_3_^−^ levels observed at REEs-rich points. In parallel, transition metals show a dual role: moderate concentrations of Mn can serve as electron acceptors, coupling iron or manganese reduction with nitrate reduction, while excessive levels inhibit microbial metabolism, attenuating their contribution to denitrification [29,30]. Similarly, Cu and Zn act as cofactors for nitrification and denitrification enzymes at trace levels [31], but in excess, they disrupt thiol-containing active sites (e.g., the cysteine in the active site and the second site of caspase 8; active-site cysteine residues), impairing enzymatic turnover [32,33]. Collectively, these findings indicate that REE–metal interactions reconfigure aquatic biogeochemical processes by shifting the balance nitrification, and denitrification.

### 3.3. Bacterial Community Responses Based on Gene Annotation

Metagenomic sequencing and gene-based annotation revealed clear evolutions in bacterial community structure under mining-related pollution. At the control point (P1), high richness (2472–2483.5), Chao1 (2609–2628.8), ACE (2585–2591.4), Shannon (5.075–5.103) and Simpson (0.975) (Table S3) indicated a diverse and stable community. In contrast, diversity declined significantly at near-source points (P2–P3), with Shannon and Simpson indices at P3-0.2 m dropping to 3.963 and 0.946, respectively, reflecting dominance of pollution-tolerant taxa and reduced community evenness under high nitrogen, and metals and REEs stress [34]. Similar patterns were observed in 1.0 m deeper waters (P3: Shannon 4.047, Simpson 0.950), indicating vertical impacts of mining discharge. Downstream points (P4–P5) showed rise, with P5-1.0 m displaying high diversity (Shannon 5.321; Simpson 0.985), consistent with self-purification effects along the route. However, variability at P4 (Shannon 0.752–5.266; Simpson 0.752–0.980) suggests spatial heterogeneity in rise under 0.2 m and 1.0 m depth. Overall, pollution altered microbial diversity and evenness, likely restructuring functional potential in nitrogen, and organic matter cycling, with implications for ecosystem services.

Microbe-mediated denitrification can promote nitrogen loss in mining soils contaminated with rare earth elements [35]. The phylum-level analysis revealed that nitrogen-cycling microorganisms exhibited pronounced shifts in response to REEs and heavy metals along the route (Figure S7). *Pseudomonadota* dominated across all points (75% in relative abundance) and reached particularly high levels at upstream polluted points P2 and P3 (92.15–94.20%), highlighting their potential role in denitrification [36]; meanwhile, *Pseudomonadota* also adapt trace metal pollutant stress [37]. Although the surface and subsurface water at all points exhibited high ORP and was macroscopically aerobic, localized microenvironments—such as within suspended particles or biofilms—may develop oxygen gradients, creating low-oxygen niches where facultative anaerobic *Pseudomonadota* (e.g., *Pseudomonas* genus) can perform denitrification [38]. The enrichment of *Bacteroidota* at these points (2.57–3.63% at P2–P3) might be indirectly support nitrate reduction by decomposing complex organic matter and releasing low-molecular-weight carbon sources that serve as electron donors for denitrifiers [39,40,41,42]. Nevertheless, under largely aerobic, nitrate-dominated conditions, actual denitrification rates are likely constrained, and further verification using functional gene analysis or isotope-tracing experiments is needed to confirm these potential contributions in the future. Meanwhile, metagenomic sequencing revealed that metal resistance genes, including *czc*, *cnr*, *cus*, *cop*, *mer*, *chr*, and *ars* [43], were present in members of *Bacteroidota*, *Planctomycetota*, and *Myxococcota* (Table S4). In contrast, several groups with weaker tolerance, including *Planctomycetota* and *Myxococcota* [44], showed decreased abundance at P2–P3 but recovered downstream (P4–P5), indicating their sensitivity to metal stress and gradual reactivation as concentrations declined. Some studies have also shown that REEs can reduce the nitrifier activity [45]. Both taxa are involved in partial denitrification and dissimilatory nitrate reduction to ammonium (DNRA) [36,46], suggesting that mining pollution may transiently suppress nitrogen-cycling functions before partial recovery downstream. Importantly, nitrifying lineages displayed distinct responses: Nitrososphaerota (ammonia-oxidizing archaea, AOA) increased in abundance at P2 (0.14–0.16%), meaning that ammonia oxidation was promoted [47]. Similarly, *Acidobacteriota* play an important role in nitrogen cycling, though low in abundance, showed slight enrichment of 0.22–0.45% at polluted sites (P2–P3), suggesting adaptive potential in nitrogen/metal-rich environments [48]. Downstream, *Nitrospirota* contribute to aerobic nitrification, which exhibited marked recovery and expansion, consistent with their dual role in nitrification and denitrification and their capacity to thrive under reduced contaminant pressure [49,50]. At trace levels, metals function as cofactors in key redox enzymes [51], thereby stimulating nitrification and sustaining AOA and *Nitrospirota* activity. However, excessive metals concentrations, coupled with high levels of toxic metals such as Pb^2+^ and Cd^2+^, impose oxidative and membrane stress, suppressing sensitive taxa and nitrification, and reducing overall microbial diversity [52,53]. Heavy metals such as Fe and Mn exerted additional bidirectional effects: acting as alternative electron acceptors to couple with nitrate reduction at moderate concentrations, but inhibiting microbial metabolism at high levels [54]. The result shows the observed accumulation of nitrate-related microbes in REE-rich zones (P2–P3) and its partial attenuation downstream. Such patterns highlight both the resilience and vulnerability of aquatic nitrogen cycling under combined REE and heavy metal stress.

**Figure 2:**
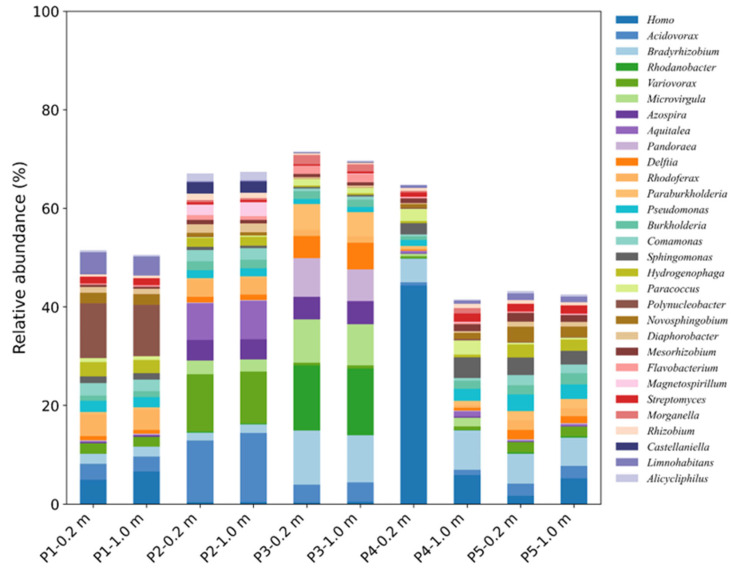
The relative abundance of top 30 genus microbial at different depths across the sampling points in the mining area.

Microbial communities in REE- and metal-rich waters exhibited pronounced restructuring, particularly among taxa mediating nitrogen cycling (Figure 2). Some microbial genes (e.g., *Acidovorax*, *Variovorax* and *Rhodoferax*) were associated with nitrogen cycle metabolism, including *nar*, *nor*, *nir*, *nxr*, and *pmoC-amoC* (Table S5) [55]. Canonical denitrifiers (*Acidovorax* [56], *Bradyrhizobium*, *Burkholderia*, *Flavobacterium* [57]) coexisted with heterotrophic nitrification–aerobic denitrification (HNAD) taxa (*Pseudomonas*, *Paracoccus*, *Streptomyces* [57], *Comamonas* [58], *Azospira* [59], *Microvirgula* [60], *Diaphorobacter* [61], *Rhizobium* [62]). Increased abundances of *Bradyrhizobium* (1.62–9.60% at P2–P3) and *Rhodanobacter* (0.19–13.51% at P2–P3) suggest that conventional denitrification remains active [63], meanwhile the frequent occurrence of HNAD genera points to a metabolic shift towards flexible pathways that sustain nitrogen removal under stress. At low concentrations, metals such as Fe, and Pb can enhance nitrate and nitrite reduction in genus such as *Acidovorax* and in genes such as *narG*, and *nirS*, and high concentration is the opposite [64,65]. Enrichment of *Variovorax* with highest 10.62–11.64% relative abundance at P2, which combine the presence of denitrification-related genes (*narK***,** *nrtP***,** and *nasA*) (Table S5) [66] and metal resistance genes (e.g., *ars*, *chr*, *cnr*, *cop*, *cus*, *czc*, *mer*, and *znt*) (Table S4), indicates their central role under stress [67]. Other taxa contributed complementary functions, such as, *Paraburkholderia* maintained nitrogen fixation, reaching its highest relative abundance at P3 (4.86–5.21%) [68], whereas *Delftia* (4.52–5.37% at P3), a denitrifying phosphate-accumulating organism, coupled denitrification [69], linking N cycling. Vertical patterns revealed further ecological partitioning, which reflects contrasting responses to redox and metal stress. Bottom layers (1.0 m) were enriched in traditional denitrifiers (*Acidovorax*, *Bradyrhizobium*). The exceedingly abundance of *Rhodanobacter* at P3 (>13%) highlights its role as a core denitrifier in highly contaminated zones [70]. These results indicate that nitrogen cycling in REE- and metal-rich waters is maintained by two processes: (i) selective filtering, enriching tolerant denitrifiers (such as, *Acidovorax, Variovorax*); and (ii) metabolic reprogramming, favoring heterotrophic nitrification and aerobic denitrification (HNAD) taxa (e.g., *Pseudomonas*, *Comamonas*, *Azospira*) that sustain nitrification–denitrification under exogenous stress. Rather than relying solely on classical denitrification, nitrogen turnover emerges from a flexible and redundant network of metal-tolerant denitrifiers, HNAD bacteria, and nitrogen-fixers.

### 3.4. KEGG Functional Gene Response Characteristics

**Figure 3:**
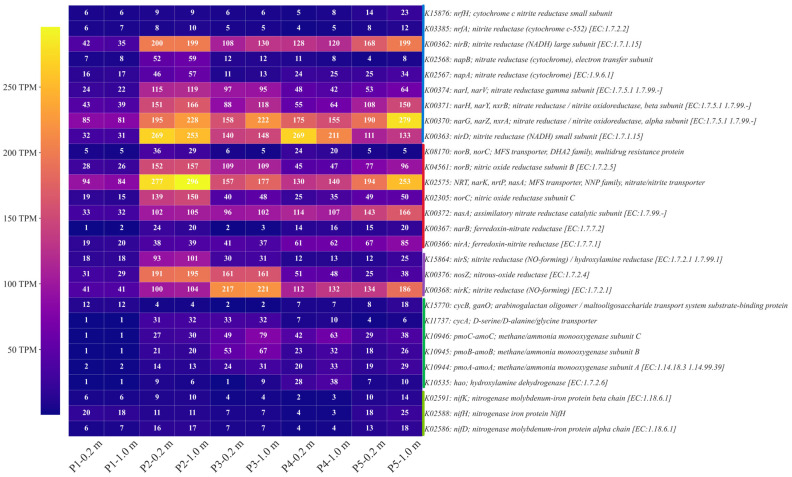
Expression profiles of KEGG functional genes involved in nitrogen metabolism. Vertical bars in blue, red, purple, green, and light green represent genes associated with DNRA, denitrification, other key nitrogen reductases, nitrification, and nitrogen fixation, respectively.

During nitrogen cycling, distinct microbial groups coordinate specific metabolic pathways and functional genes to mediate the transformation of nitrogen species, driving the dynamic transitions from NH_4_^+^−N, and NO_3_^−^−N to gaseous forms (N_2_, N_2_O, NO) [71]. KEGG-based gene annotation revealed pronounced spatial variations in the abundance of key nitrogen-cycling genes, highlighting the ecological regulation of nitrogen transformations under the combined stress of REEs and heavy metals in this mining area [72]. For example, the gene K15876, corresponding to *nrfH* and encoding the cytochrome c nitrite reductase small subunit (Figure 3), is central to the DNRA pathway, where it functions in concert with *nrfA* to catalyze the reduction of nitrite to NH_4_^+^ [73]. Its content was highest at point P5-1.0 m (23 TPM) compared with the P1 (6 TPM), indicating a substantial enhancement of DNRA across the route. Correspondingly, K03385 (nrfA, nitrite reductase) was also relatively high at P5-1.0 m (12 TPM), further supporting the activity of the DNRA pathway. This finding suggests that, in the water bodies of the mining area, the microbial community is more likely to conserve nitrogen through DNRA rather than release it entirely in gaseous form. Nitrate reduction in these systems proceeds mainly through two pathways: the membrane-bound type (*Nar*) and the periplasmic type (*Nap*) [74,75]. K00363 (*nirD*), K00370 (*narG*, *narZ*, *nxrA*), K00371 (*narH*, *narY*, *nxrB*), and K00374 (*narI*, *narV*) enriched at P2 (e.g., K00363 at P2 m reached 253–269 TPM, compared with 31–32 TPM at P1), indicating enhanced nitrate-to-nitrite reduction capacity in these zones. Similarly, the periplasmic K02567 (*napA*) and K02568 (*napB*) exhibited high contents at P2 (46–57 and 52–59 TPM, respectively), suggesting that bacteria at this point efficiently use nitrate as an electron acceptor, and help to drive anaerobic metabolism [76]. In the subsequent step of nitrite reduction, the major pathways involve K15864 (*nirS*)/ K00368 (*nirK*) (NO_2_^−^ → NO), K08170 (*norB/norC*)/ K04561 (*norB*)/ K02305 (*norC*) (NO → N_2_O), and K00376 (*nosZ*) (N_2_O → N_2_) [77]. K00368 (*nirK*) content was particularly high in the P3 (0.2 m, 217 TPM; 1.0 m, 221 TPM), while K15864 (*nirS*) reached 93 TPM at P2-0.2 m and 101 TPM at P2-1.0 m, indicating increased activity of nitrite reduction at these points. Consistently, *norB-* and *norC*-related KEGG genes were enriched at P2, suggesting a complete NO-to-N_2_O conversion chain. Although *nosZ* was also abundant at P2 and P3 (191–195 and 161 TPM, respectively), its level declined sharply at P5 (25–38 TPM), implying a potential risk of N_2_O accumulation downstream. Shu et al. reported that rivers impacted by ion-adsorption rare earth mining in Yangtze River, China, exhibit N_2_O emissions [78]. Given that N_2_O is a potent greenhouse gas, this pattern indicates that the terminal environment of the mining area may shift from “complete denitrification” to “partial denitrification,” thereby altering the fate of nitrogen. Furthermore, nitrogenase genes K02591 (*nifK*), K02588 (*nifH*), and K02586 (*nifD*) were enriched at P5 (e.g., *nifH* = 25 TPM at P5-1.0 m vs. 7 TPM at P3-1.0 m), implying activation of nitrogen fixation at downstream [79]. Correspondingly, ammonia monooxygenases (K10944 amoA, K10945 amoB, K10946 amoC) and hydroxylamine dehydrogenase (K10535 hao) exhibited strong activity at P3–P4, reflecting alternating dominance of nitrification, denitrification, DNRA, and nitrogen fixation across points [80]. Collectively, these results indicate that the nitrogen cycle in the mining area is a dynamic balance among multiple pathways—nitrification, denitrification, DNRA, and nitrogen fixation—modulated by spatial distribution and environmental stress.

## 4. Conclusions

Integrated analyses of physicochemical parameters, metals, REEs, and metagenomes revealed pronounced spatial gradients of contamination in mining waters. Near-source points (P2–P3) exhibited high nitrogen, metal, and REE accumulation, reduced microbial diversity, and enrichment of denitrification- and metal-resistance genes, whereas downstream points (P4–P5) showed partial recovery, reflecting self-purification. Importantly, functional gene annotation indicated incomplete denitrification, with reduced *nosZ* downstream and a potential risk of N_2_O accumulation. Nitrogen turnover was maintained not only by classical denitrifiers (*Acidovorax*, *Bradyrhizobium*, *Rhodanobacter*) but also by HNAD taxa (*Pseudomonas*, *Comamonas*, *Azospira*) and nitrogen fixers (*Paraburkholderia*), forming a flexible and redundant microbial network under stress. These findings highlight the dual processes of selective filtering of tolerant denitrifiers and metabolic reprogramming toward HNAD that sustain nitrogen cycling under combined REE and heavy metal pressures. Meanwhile, these findings highlight the need to prioritize monitoring and management at discharge sources and downstream sections, combining measures such as wastewater treatment, pollutant interception, and ecological restoration to prevent long-term accumulation and ecosystem degradation.

Future work should integrate metabolomics, and ecological modeling to elucidate microbial functional transfer and pollutant migration under high contamination pressure. Continuous monitoring of rare earths and specific metals may further serve as sensitive indicators for ecological risk, supporting precision strategies for water quality management. Overall, this study provides a systematic assessment of mining-related water pollution, microbial responses, and ecological risks, offering both theoretical and practical insights for sustainable water management in mining regions.

## Figures and Tables

**Table 1:** TN, NH_4_^+^−N, NO_3_^−^−N, and SO_4_^2−^ contents at different depths across the sampling points.

	TN Content (mg/L)		NH_4_^+^−N (mg/L)		NO_3_^−^−N (mg/L)		SO_4_^2−^ (mg/L)	
Depths	0.2 m	1.0 m	0.2 m	1.0 m	0.2 m	1.0 m	0.2 m	1.0 m
P1	5.0 ± 0.19	5.2 ± 0.30	0.4 ± 0.01	0.4 ± 0.01	6.1 ± 0.05	6.5 ± 0.11	232.0 ± 3.81	249.7 ± 1.89
P2	88.2 ± 4.13	90.7 ± 2.26	4.9 ± 0.06	5.1 ± 0.02	89.7 ± 0.54	100.7 ± 0.37	62.6 ± 0.56	63.3 ± 0.63
P3	103.4 ± 2.75	102.6 ± 3.96	4.2 ± 0.01	4.3 ± 0.01	104.1 ± 0.20	107.7 ± 0.56	123.4 ± 2.17	124.7 ± 1.46
P4	22.4 ± 1.97	28.0 ± 1.77	4.2 ± 0.02	4.1 ± 0.02	29.4 ± 0.52	36.6 ± 0.55	96.7 ± 1.10	122.6 ± 1.92
P5	1.9 ± 0.08	8.2 ± 0.68	0.2 ± 0.01	0.1 ± 0.00	2.3 ± 0.04	13.0 ± 0.19	17.0 ± 0.62	17.4 ± 0.97

## Data Availability

The original contributions presented in this study are included in the article/Supplementary Material. Further inquiries can be directed to the corresponding author.

## Supplementary Materials

The following supporting information can be downloaded at https://www.mdpi.com/article/10.3390/microorganisms13102236/s1, Figure S1: COD contents at different depths across the sampling points; Figure S2: DO contents at different depths across the sampling points; Figure S3: ORP of water at different depths across the sampling points; Figure S4: F^−^ contents at different depths across the sampling points; Figure S5: Water temperature at different depths across the sampling points; Figure S6: pH value at different depths across the sampling points; Figure S7: The relative abundance of top 20 phylum microbial at different depths across the sampling points; Table S1: Metal contents (mg/L) at different depths across the sampling points; Table S2: Rare earth elements contents (mg/L) at different depths across the sampling points; Table S3: Alpha diversity index at the genus level; Table S4: Relationships between the top 30 microbial genera (Figure 2), their corresponding phyla, and metal resistance genes; Table S5: Relationships between the top 30 microbial genera (Figure 2), their corresponding phyla, and KEGG functional genes (Figure 3) involved in nitrogen metabolism.
