# Supplementary material for: Ecological Imprint of Rare Earth Mining on Microbial Communities and Water Quality Across Depth and Distance Gradients in Ganzhou, China
Source: Microorganisms. 2025 Sep 24;13(10):2236. doi: 10.3390/microorganisms13102236 (PMC12566276; doi:10.3390/microorganisms13102236)
Supplement: Supplementary file 1 [file microorganisms-13-02236-s001.zip › microorganisms-3855889-supplementary.pdf]

Article

Ecological Imprint of Rare Earth Mining on Microbial Communities and Water Quality Across Depth and Distance Gradients in Ganzhou, China

Yian Wang [1], Fei Shi [1], Fengxiang Lang [2], Guohua Wang [1], Yan Mao [2], Yingjie Xiao [2], Li Yin [1], Genhe He [1] and Yonghui Liao [1,*]

[1] Key Laboratory of Jiangxi Province for Functional Biology and Pollution Control in Red Soil Regions, School of Life Sciences, Jinggangshan University, Ji'an 343000, China; nickowya@163.com (Y.W.)
[2] Hydrology and Water Resources Monitoring Center of the Middle Reaches of Ganjiang River, Ji'an 343000, China
* Correspondence: liaoyonghui1104@126.com; Tel.: +86-15951813018

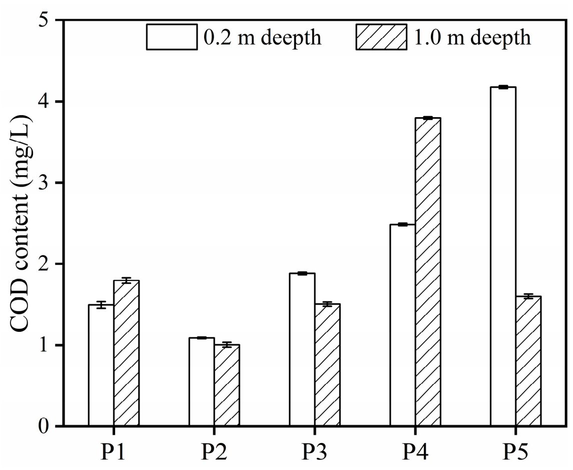

Figure S1. COD contents at different depths across the sampling points.

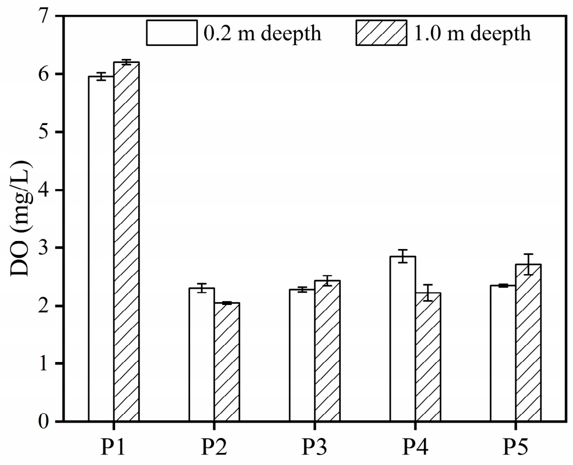

Figure S2. DO contents at different depths across the sampling points.

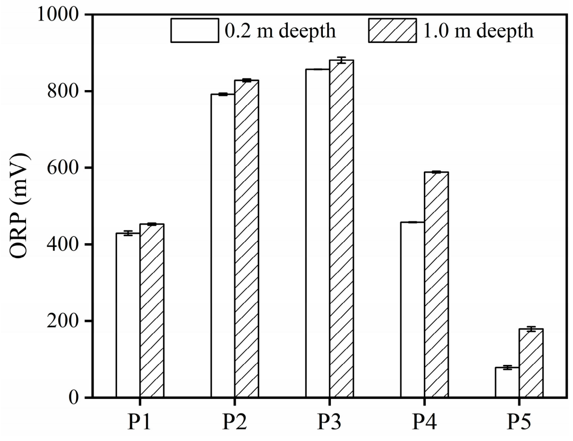

Figure S3. ORP of water at different depths across the sampling points.

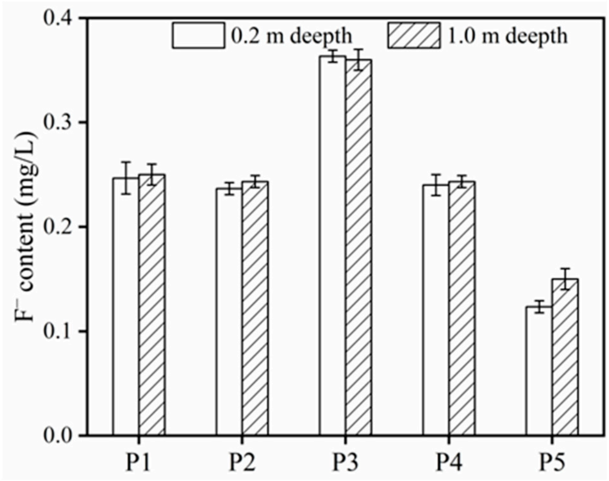

Figure S4. F^- contents at different depths across the sampling points.

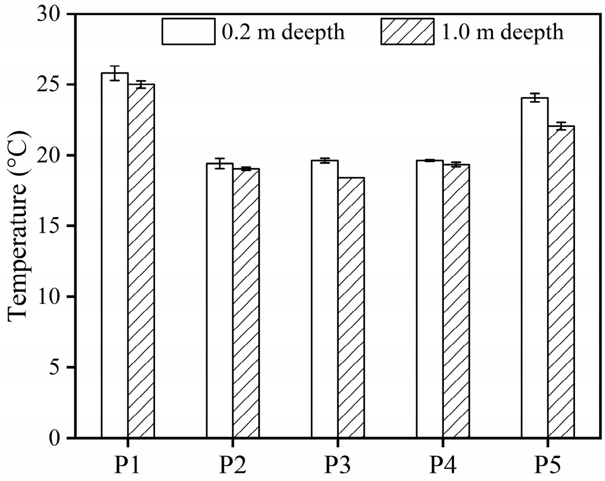

Figure S5. Water temperature at different depths across the sampling points.

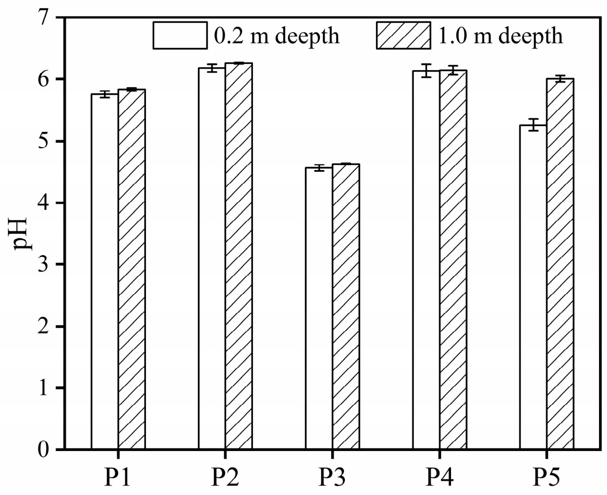

Figure S6. pH value at different depths across the sampling points.

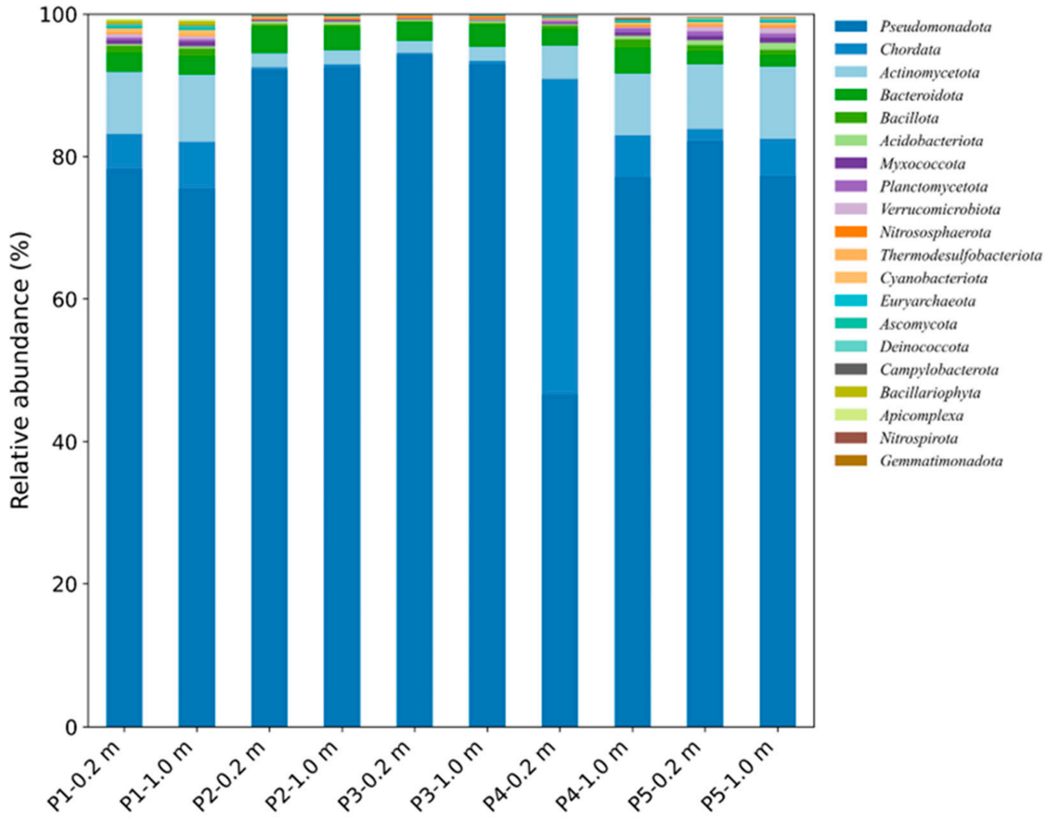

Figure S7. The relative abundance of top 20 phylum microbial at different depths across the sampling points.

Table S1. Metal contents (mg/L) at different depths across the sampling points.

	As	Fe	Mn	Cu	Zn	Pb	Cd	Cr
P1-0.2 m	0.001	<0.03	0.347	0.004	0.063	0.004	0.020	<0.004
STDEV	0.000		0.006	0.001	0.001	0.001	0.000	
P1-1.0 m	0.001	−	0.447	0.003	0.058	0.132	0.002	−
STDEV	0.000		0.006	0.001	0.000	0.001	0.000	
P2-0.2 m	0.001	−	2.127	<0.002	0.363	<0.004	0.002	−
STDEV	0.000		0.015		0.000		0.000	
P2-1.0 m	0.001	−	2.347	−	0.375	0.004	0.003	−
STDEV	0.000		0.015		0.000	0.001	0.000	
P3-0.2 m	0.001	−	3.367	0.051	0.494	0.195	0.005	−
STDEV	0.000		0.051	0.002	0.000	0.002	0.000	
P3-1.0 m	0.001	−	3.393	0.038	0.535	0.025	0.007	−
STDEV	0.000		0.032	0.001	0.000	0.001	0.000	
P4-0.2 m	0.001	<0.03	1.443	<0.002	0.502	0.035	0.043	<0.004
STDEV	0.000		0.025		0.000	0.002	0.000	
P4-1.0 m	0.001	−	0.613	0.002	0.647	0.017	0.018	−
STDEV	0.000		0.025	0.001	0.000	0.001	0.000	
P5-0.2 m	0.001	0.117	0.253	0.006	0.039	0.048	0.001	−
STDEV	0.000	0.006	0.015	0.001	0.001	0.002	0.000	
P5-1.0 m	0.001	<0.03	0.147	0.003	0.023	0.020	0.004	−
STDEV	0.000		0.006	0.001	0.000	0.002	0.000	

Note: − represents below the detection line

Table S2. Rare earth elements contents (mg/L) at different depths across the sampling points.

	La	Ce	Pr	Nd	Sm	Eu	Gd	Tb	Dy	Ho	Er	Tm	Yb	Lu	Sc	Y
P1-0.2 m	0.020	0.010	ND	0.006	0.001	ND	0.003	ND	0.002	ND	0.000	0.001	0.000	0.000	0.000	0.009
P1-1.0 m	0.024	0.013	ND	0.007	0.002	ND	0.002	ND	0.003	ND	0.000	ND	0.000	0.000	0.000	0.016
P2-0.2 m	0.173	0.055	0.022	0.082	0.011	0.001	0.011	ND	0.025	0.001	0.003	0.007	0.004	0.000	0.000	0.088
P2-1.0 m	0.208	0.059	0.015	0.089	0.010	0.001	0.017	ND	0.029	0.002	0.007	0.005	0.004	0.000	0.000	0.113
P3-0.2 m	0.982	0.189	0.154	0.644	0.143	0.018	0.116	0.002	0.152	0.017	0.044	0.022	0.033	0.004	0.000	0.537
P3-1.0 m	1.063	0.201	0.169	0.691	0.157	0.019	0.124	0.001	0.160	0.018	0.047	0.027	0.035	0.004	0.000	0.588
P4-0.2 m	0.052	0.007	0.003	0.030	0.007	ND	0.003	ND	0.006	ND	0.002	0.002	0.000	0.000	0.000	0.014
P4-1.0 m	0.040	0.011	0.007	0.037	0.008	ND	0.005	ND	0.007	ND	0.001	0.006	0.000	0.000	0.000	0.030
P5-0.2 m	0.013	0.018	0.005	0.013	0.003	ND	0.002	ND	0.002	0.000	0.001	ND	0.000	0.000	0.000	0.007
P5-1.0 m	0.004	0.016	0.002	0.009	0.001	ND	0.000	ND	0.002	ND	0.001	ND	0.000	0.000	0.000	0.005

Table S3. Alpha diversity index at the genus level

	P1-0.2 m	P1-1.0 m	P2-0.2 m	P2-1.0 m	P3-0.2 m	P3-1.0 m	P4-0.2 m	P4-1.0 m	P5-0.2 m	P5-1.0 m
Richness	2472.0±12.7	2483.5±7.8	2186.0±8.5	2201.5±6.4	2083.5±2.1	2111.0±7.1	2269.0±117.4	2358.0±17.0	2422.0±4.2	2396.5±3.5
Chao1	2609.0±41.8	2628.8±54.5	2310.4±13.5	2331.8±50.8	2193.2±16.7	2238.8±14.2	2398.8±145.5	2493.7±34.2	2555.2±35.1	2528.5±9.5
ACE	2585.3±35.0	2591.4±31.3	2281.3±20.2	2303.6±28.7	2174.3±8.9	2201.5±11.8	2348.5±134.7	2453.0±48.0	2524.7±21.4	2501.5±1.2
Shannon	5.075±0.011	5.103±0.091	4.346±0.002	4.307±0.008	3.963±0.010	4.047±0.001	3.804±2.190	5.266±0.173	5.290±0.003	5.321±0.053
Simpson	0.975±0.001	0.975±0.004	0.957±0.000	0.955±0.000	0.946±0.000	0.950±0.000	0.752±0.327	0.980±0.006	0.986±0.000	0.985±0.002

Table S4. Relationships between the top 30 microbial genera (**Figure 2**), their corresponding phyla, and metal resistance genes

**Phylum**	**Genus**	**KO**	**Genes**
Pseudomonadota	*Acidovorax*	K03325	*ACR3, arsB*
Pseudomonadota	*Aquitalea*	K03325	*ACR3, arsB*
Pseudomonadota	*Bradyrhizobium*	K03325	*ACR3, arsB*
Pseudomonadota	*Burkholderia*	K03325	*ACR3, arsB*
Pseudomonadota	*Limnohabitans*	K03325	*ACR3, arsB*
Pseudomonadota	*Magnetospirillum*	K03325	*ACR3, arsB*
Pseudomonadota	*Microvirgula*	K03325	*ACR3, arsB*
Pseudomonadota	*Polynucleobacter*	K03325	*ACR3, arsB*
Pseudomonadota	*Pseudomonas*	K03325	*ACR3, arsB*
Pseudomonadota	*Rhodoferax*	K03325	*ACR3, arsB*
Pseudomonadota	*Alicycliphilus*	K01551	*arsA, ASNA1, GET3*
Pseudomonadota	*Comamonas*	K01551	*arsA, ASNA1, GET3*
Pseudomonadota	*Magnetospirillum*	K01551	*arsA, ASNA1, GET3*
Pseudomonadota	*Rhizobium*	K01551	*arsA, ASNA1, GET3*
Pseudomonadota	*Rhodoferax*	K01551	*arsA, ASNA1, GET3*
Pseudomonadota	*Variovorax*	K01551	*arsA, ASNA1, GET3*
Pseudomonadota	*Rhizobium*	K03893	*arsB*
Pseudomonadota	*Variovorax*	K03893	*arsB*
Pseudomonadota	*Acidovorax*	K03741	*arsC*
Pseudomonadota	*Acidovorax*	K00537	*arsC*
Pseudomonadota	*Aquitalea*	K03741	*arsC*
Pseudomonadota	*Aquitalea*	K00537	*arsC*
Pseudomonadota	*Bradyrhizobium*	K03741	*arsC*
Pseudomonadota	*Bradyrhizobium*	K00537	*arsC*
Pseudomonadota	*Comamonas*	K03741	*arsC*
Bacteroidota	*Flavobacterium*	K03741	*arsC*
Pseudomonadota	*Limnohabitans*	K03741	*arsC*
Pseudomonadota	*Magnetospirillum*	K03741	*arsC*
Pseudomonadota	*Magnetospirillum*	K00537	*arsC*
Pseudomonadota	*Microvirgula*	K03741	*arsC*
Pseudomonadota	*Morganella*	K00537	*arsC*
Pseudomonadota	*Novosphingobium*	K03741	*arsC*
Pseudomonadota	*Novosphingobium*	K00537	*arsC*
Pseudomonadota	*Polynucleobacter*	K03741	*arsC*
Pseudomonadota	*Rhizobium*	K03741	*arsC*
Pseudomonadota	*Rhizobium*	K00537	*arsC*
Pseudomonadota	*Rhodoferax*	K03741	*arsC*
Pseudomonadota	*Rhodoferax*	K00537	*arsC*
Pseudomonadota	*Sphingomonas*	K00537	*arsC*

Pseudomonadota	*Variovorax*	K03741	*arsC*
Pseudomonadota	*Variovorax*	K00537	*arsC*
Pseudomonadota	*Bradyrhizobium*	K11811	*arsH*
Pseudomonadota	*Burkholderia*	K11811	*arsH*
Pseudomonadota	*Novosphingobium*	K11811	*arsH*
Pseudomonadota	*Rhizobium*	K11811	*arsH*
Pseudomonadota	*Rhodoferax*	K11811	*arsH*
Pseudomonadota	*Variovorax*	K11811	*arsH*
Pseudomonadota	*Acidovorax*	K03892	*arsR*
Pseudomonadota	*Aquitalea*	K03892	*arsR*
Pseudomonadota	*Azospira*	K03892	*arsR*
Pseudomonadota	*Bradyrhizobium*	K03892	*arsR*
Pseudomonadota	*Burkholderia*	K03892	*arsR*
Pseudomonadota	*Comamonas*	K03892	*arsR*
Pseudomonadota	*Limnohabitans*	K03892	*arsR*
Pseudomonadota	*Magnetospirillum*	K03892	*arsR*
Pseudomonadota	*Novosphingobium*	K03892	*arsR*
Pseudomonadota	*Polynucleobacter*	K03892	*arsR*
Pseudomonadota	*Rhizobium*	K03892	*arsR*
Pseudomonadota	*Rhodoferax*	K03892	*arsR*
Pseudomonadota	*Acidovorax*	K23988	*arsR-arsC*
Pseudomonadota	*Acidovorax*	K07213	*ATOX1, ATX1, copZ, golB*
Pseudomonadota	*Castellaniella*	K07213	*ATOX1, ATX1, copZ, golB*
Pseudomonadota	*Polynucleobacter*	K07213	*ATOX1, ATX1, copZ, golB*
Pseudomonadota	*Variovorax*	K07213	*ATOX1, ATX1, copZ, golB*
Pseudomonadota	*Aquitalea*	K07240	*chrA*
Pseudomonadota	*Azospira*	K07240	*chrA*
Pseudomonadota	*Bradyrhizobium*	K07240	*chrA*
Pseudomonadota	*Burkholderia*	K07240	*chrA*
Bacteroidota	*Flavobacterium*	K07240	*chrA*
Pseudomonadota	*Limnohabitans*	K07240	*chrA*
Pseudomonadota	*Magnetospirillum*	K07240	*chrA*
Pseudomonadota	*Novosphingobium*	K07240	*chrA*
Pseudomonadota	*Polynucleobacter*	K07240	*chrA*
Pseudomonadota	*Pseudomonas*	K07240	*chrA*
Pseudomonadota	*Rhizobium*	K07240	*chrA*
Pseudomonadota	*Rhodoferax*	K07240	*chrA*
Pseudomonadota	*Variovorax*	K07240	*chrA*
Pseudomonadota	*Bradyrhizobium*	K07167	*chrR*
Pseudomonadota	*Mesorhizobium*	K07167	*chrR*
Pseudomonadota	*Acidovorax*	K19784	*chrR, NQR*
Pseudomonadota	*Aquitalea*	K19784	*chrR, NQR*
Pseudomonadota	*Castellaniella*	K19784	*chrR, NQR*

Pseudomonadota	*Magnetospirillum*	K19784	*chrR, NQR*
Pseudomonadota	*Microvirgula*	K19784	*chrR, NQR*
Pseudomonadota	*Novosphingobium*	K19784	*chrR, NQR*
Pseudomonadota	*Paraburkholderia*	K19784	*chrR, NQR*
Pseudomonadota	*Pseudomonas*	K19784	*chrR, NQR*
Pseudomonadota	*Rhizobium*	K19784	*chrR, NQR*
Pseudomonadota	*Rhodanobacter*	K19784	*chrR, NQR*
Pseudomonadota	*Rhodoferax*	K19784	*chrR, NQR*
Pseudomonadota	*Sphingomonas*	K19784	*chrR, NQR*
Pseudomonadota	*Variovorax*	K19784	*chrR, NQR*
Pseudomonadota	*Novosphingobium*	K24073	*cnrH*
Pseudomonadota	*Novosphingobium*	K24078	*cnrR, cnrX*
Pseudomonadota	*Acidovorax*	K17686	*copA, ctpA, ATP7*
Pseudomonadota	*Bradyrhizobium*	K17686	*copA, ctpA, ATP7*
Pseudomonadota	*Burkholderia*	K17686	*copA, ctpA, ATP7*
Pseudomonadota	*Comamonas*	K17686	*copA, ctpA, ATP7*
Bacteroidota	*Flavobacterium*	K17686	*copA, ctpA, ATP7*
Pseudomonadota	*Hydrogenophaga*	K17686	*copA, ctpA, ATP7*
Pseudomonadota	*Limnohabitans*	K17686	*copA, ctpA, ATP7*
Pseudomonadota	*Magnetospirillum*	K17686	*copA, ctpA, ATP7*
Pseudomonadota	*Novosphingobium*	K17686	*copA, ctpA, ATP7*
Pseudomonadota	*Paracoccus*	K17686	*copA, ctpA, ATP7*
Pseudomonadota	*Polynucleobacter*	K17686	*copA, ctpA, ATP7*
Pseudomonadota	*Rhizobium*	K17686	*copA, ctpA, ATP7*
Pseudomonadota	*Rhodoferax*	K17686	*copA, ctpA, ATP7*
Pseudomonadota	*Sphingomonas*	K17686	*copA, ctpA, ATP7*
Pseudomonadota	*Variovorax*	K17686	*copA, ctpA, ATP7*
Pseudomonadota	*Acidovorax*	K01533	*copB*
Pseudomonadota	*Bradyrhizobium*	K01533	*copB*
Bacteroidota	*Flavobacterium*	K01533	*copB*
Pseudomonadota	*Hydrogenophaga*	K01533	*copB*
Pseudomonadota	*Limnohabitans*	K01533	*copB*
Pseudomonadota	*Magnetospirillum*	K01533	*copB*
Pseudomonadota	*Mesorhizobium*	K01533	*copB*
Pseudomonadota	*Novosphingobium*	K01533	*copB*
Pseudomonadota	*Polynucleobacter*	K01533	*copB*
Pseudomonadota	*Rhodoferax*	K01533	*copB*
Pseudomonadota	*Sphingomonas*	K01533	*copB*
Pseudomonadota	*Variovorax*	K01533	*copB*
Pseudomonadota	*Aquitalea*	K07156	*copC, pcoC*
Pseudomonadota	*Burkholderia*	K07156	*copC, pcoC*
Pseudomonadota	*Magnetospirillum*	K07156	*copC, pcoC*
Pseudomonadota	*Novosphingobium*	K07156	*copC, pcoC*

Pseudomonadota	*Acidovorax*	K07665	*cusR, copR, silR*
Pseudomonadota	*Azospira*	K07665	*cusR, copR, silR*
Pseudomonadota	*Burkholderia*	K07665	*cusR, copR, silR*
Pseudomonadota	*Castellaniella*	K07665	*cusR, copR, silR*
Pseudomonadota	*Magnetospirillum*	K07665	*cusR, copR, silR*
Pseudomonadota	*Microvirgula*	K07665	*cusR, copR, silR*
Pseudomonadota	*Rhodanobacter*	K07665	*cusR, copR, silR*
Pseudomonadota	*Rhodoferax*	K07665	*cusR, copR, silR*
Pseudomonadota	*Variovorax*	K07665	*cusR, copR, silR*
Pseudomonadota	*Acidovorax*	K07644	*cusS, copS, silS*
Pseudomonadota	*Alicycliphilus*	K07644	*cusS, copS, silS*
Pseudomonadota	*Bradyrhizobium*	K07644	*cusS, copS, silS*
Pseudomonadota	*Burkholderia*	K07644	*cusS, copS, silS*
Pseudomonadota	*Castellaniella*	K07644	*cusS, copS, silS*
Pseudomonadota	*Limnohabitans*	K07644	*cusS, copS, silS*
Pseudomonadota	*Magnetospirillum*	K07644	*cusS, copS, silS*
Pseudomonadota	*Paraburkholderia*	K07644	*cusS, copS, silS*
Pseudomonadota	*Pseudomonas*	K07644	*cusS, copS, silS*
Pseudomonadota	*Rhodanobacter*	K07644	*cusS, copS, silS*
Pseudomonadota	*Rhodoferax*	K07644	*cusS, copS, silS*
Pseudomonadota	*Sphingomonas*	K07644	*cusS, copS, silS*
Pseudomonadota	*Variovorax*	K07644	*cusS, copS, silS*
Pseudomonadota	*Acidovorax*	K15726	*czcA, cusA, cnrA*
Pseudomonadota	*Alicycliphilus*	K15726	*czcA, cusA, cnrA*
Pseudomonadota	*Bradyrhizobium*	K15726	*czcA, cusA, cnrA*
Pseudomonadota	*Burkholderia*	K15726	*czcA, cusA, cnrA*
Pseudomonadota	*Comamonas*	K15726	*czcA, cusA, cnrA*
Bacteroidota	*Flavobacterium*	K15726	*czcA, cusA, cnrA*
Pseudomonadota	*Limnohabitans*	K15726	*czcA, cusA, cnrA*
Pseudomonadota	*Novosphingobium*	K15726	*czcA, cusA, cnrA*
Pseudomonadota	*Paraburkholderia*	K15726	*czcA, cusA, cnrA*
Pseudomonadota	*Pseudomonas*	K15726	*czcA, cusA, cnrA*
Pseudomonadota	*Rhodoferax*	K15726	*czcA, cusA, cnrA*
Pseudomonadota	*Sphingomonas*	K15726	*czcA, cusA, cnrA*
Pseudomonadota	*Variovorax*	K15726	*czcA, cusA, cnrA*
Pseudomonadota	*Acidovorax*	K15727	*czcB, cusB, cnrB*
Pseudomonadota	*Alicycliphilus*	K15727	*czcB, cusB, cnrB*
Pseudomonadota	*Bradyrhizobium*	K15727	*czcB, cusB, cnrB*
Bacteroidota	*Flavobacterium*	K15727	*czcB, cusB, cnrB*
Pseudomonadota	*Limnohabitans*	K15727	*czcB, cusB, cnrB*
Pseudomonadota	*Magnetospirillum*	K15727	*czcB, cusB, cnrB*
Pseudomonadota	*Novosphingobium*	K15727	*czcB, cusB, cnrB*
Pseudomonadota	*Pseudomonas*	K15727	*czcB, cusB, cnrB*

Pseudomonadota	*Rhodanobacter*	K15727	*czcB, cusB, cnrB*
Pseudomonadota	*Rhodoferax*	K15727	*czcB, cusB, cnrB*
Pseudomonadota	*Sphingomonas*	K15727	*czcB, cusB, cnrB*
Actinomycetota	*Streptomyces*	K15727	*czcB, cusB, cnrB*
Pseudomonadota	*Variovorax*	K15727	*czcB, cusB, cnrB*
Pseudomonadota	*Acidovorax*	K15725	*czcC, cusC, cnrC*
Pseudomonadota	*Alicycliphilus*	K15725	*czcC, cusC, cnrC*
Pseudomonadota	*Azospira*	K15725	*czcC, cusC, cnrC*
Pseudomonadota	*Bradyrhizobium*	K15725	*czcC, cusC, cnrC*
Pseudomonadota	*Burkholderia*	K15725	*czcC, cusC, cnrC*
Pseudomonadota	*Comamonas*	K15725	*czcC, cusC, cnrC*
Bacteroidota	*Flavobacterium*	K15725	*czcC, cusC, cnrC*
Pseudomonadota	*Hydrogenophaga*	K15725	*czcC, cusC, cnrC*
Pseudomonadota	*Limnohabitans*	K15725	*czcC, cusC, cnrC*
Pseudomonadota	*Magnetospirillum*	K15725	*czcC, cusC, cnrC*
Pseudomonadota	*Novosphingobium*	K15725	*czcC, cusC, cnrC*
Pseudomonadota	*Pseudomonas*	K15725	*czcC, cusC, cnrC*
Pseudomonadota	*Rhodanobacter*	K15725	*czcC, cusC, cnrC*
Pseudomonadota	*Rhodoferax*	K15725	*czcC, cusC, cnrC*
Pseudomonadota	*Sphingomonas*	K15725	*czcC, cusC, cnrC*
Pseudomonadota	*Variovorax*	K15725	*czcC, cusC, cnrC*
Pseudomonadota	*Bradyrhizobium*	K16264	*czcD, zitB*
Bacteroidota	*Flavobacterium*	K16264	*czcD, zitB*
Pseudomonadota	*Hydrogenophaga*	K16264	*czcD, zitB*
Pseudomonadota	*Magnetospirillum*	K16264	*czcD, zitB*
Pseudomonadota	*Mesorhizobium*	K16264	*czcD, zitB*
Pseudomonadota	*Novosphingobium*	K16264	*czcD, zitB*
Pseudomonadota	*Rhodoferax*	K16264	*czcD, zitB*
Pseudomonadota	*Sphingomonas*	K16264	*czcD, zitB*
Pseudomonadota	*Variovorax*	K16264	*czcD, zitB*
Actinomycetota	*Streptomyces*	K00320	*mer*
Pseudomonadota	*Bradyrhizobium*	K00520	*merA*
Pseudomonadota	*Novosphingobium*	K00520	*merA*
Pseudomonadota	*Paracoccus*	K00520	*merA*
Pseudomonadota	*Pseudomonas*	K00520	*merA*
Pseudomonadota	*Rhizobium*	K00520	*merA*
Pseudomonadota	*Rhodoferax*	K00520	*merA*
Pseudomonadota	*Variovorax*	K00520	*merA*
Pseudomonadota	*Bradyrhizobium*	K19058	*merC*
Pseudomonadota	*Castellaniella*	K19058	*merC*
Pseudomonadota	*Variovorax*	K19058	*merC*
Pseudomonadota	*Acidovorax*	K08364	*merP*
Pseudomonadota	*Bradyrhizobium*	K08364	*merP*

Pseudomonadota	*Castellaniella*	K08364	*merP*
Pseudomonadota	*Comamonas*	K08364	*merP*
Pseudomonadota	*Novosphingobium*	K08364	*merP*
Pseudomonadota	*Pseudomonas*	K08364	*merP*
Pseudomonadota	*Rhodoferax*	K08364	*merP*
Pseudomonadota	*Bradyrhizobium*	K08365	*merR*
Pseudomonadota	*Burkholderia*	K08365	*merR*
Pseudomonadota	*Castellaniella*	K08365	*merR*
Pseudomonadota	*Hydrogenophaga*	K08365	*merR*
Pseudomonadota	*Novosphingobium*	K08365	*merR*
Pseudomonadota	*Polynucleobacter*	K08365	*merR*
Pseudomonadota	*Pseudomonas*	K08365	*merR*
Pseudomonadota	*Rhodoferax*	K08365	*merR*
Pseudomonadota	*Acidovorax*	K08363	*merT*
Pseudomonadota	*Bradyrhizobium*	K08363	*merT*
Pseudomonadota	*Burkholderia*	K08363	*merT*
Bacteroidota	*Flavobacterium*	K08363	*merT*
Pseudomonadota	*Novosphingobium*	K08363	*merT*
Pseudomonadota	*Rhodoferax*	K08363	*merT*
Pseudomonadota	*Castellaniella*	K07233	*pcoB, copB*
Pseudomonadota	*Novosphingobium*	K07233	*pcoB, copB*
Pseudomonadota	*Acidovorax*	K01534	*zntA*
Pseudomonadota	*Alicycliphilus*	K01534	*zntA*
Pseudomonadota	*Burkholderia*	K01534	*zntA*
Pseudomonadota	*Comamonas*	K01534	*zntA*
Bacteroidota	*Flavobacterium*	K01534	*zntA*
Pseudomonadota	*Hydrogenophaga*	K01534	*zntA*
Pseudomonadota	*Limnohabitans*	K01534	*zntA*
Pseudomonadota	*Mesorhizobium*	K01534	*zntA*
Pseudomonadota	*Novosphingobium*	K01534	*zntA*
Pseudomonadota	*Paracoccus*	K01534	*zntA*
Pseudomonadota	*Rhodoferax*	K01534	*zntA*
Pseudomonadota	*Aquitalea*	K16074	*zntB*
Pseudomonadota	*Magnetospirillum*	K16074	*zntB*
Pseudomonadota	*Rhizobium*	K16074	*zntB*
Pseudomonadota	*Rhodoferax*	K16074	*zntB*
Pseudomonadota	*Variovorax*	K16074	*zntB*

Table S5. Relationships between the top 30 microbial genera (**Figure 2**), their corresponding phyla, and KEGG functional genes (**Figure 3**) involved in nitrogen metabolism

**Phylum**	**Genus**	**KO**	**Genes**
Pseudomonadota	*Acidovorax*	K00370	*narG, narZ, nxrA*
Pseudomonadota	*Bradyrhizobium*	K02567	*napA*
Pseudomonadota	*Bradyrhizobium*	K00371	*narH, narY, nxrB*
Pseudomonadota	*Bradyrhizobium*	K00370	*narG, narZ, nxrA*
Pseudomonadota	*Bradyrhizobium*	K02575	*NRT, narK, nrtP, nasA*
Pseudomonadota	*Bradyrhizobium*	K00372	*nasA*
Pseudomonadota	*Bradyrhizobium*	K00368	*nirK*
Pseudomonadota	*Rhodanobacter*	K00362	*nirB*
Pseudomonadota	*Variovorax*	K02575	*NRT, narK, nrtP, nasA*
Pseudomonadota	*Azospira*	K00372	*nasA*
Pseudomonadota	*Rhodoferax*	K02567	*napA*
Pseudomonadota	*Rhodoferax*	K00374	*narI, narV*
Pseudomonadota	*Rhodoferax*	K00371	*narH, narY, nxrB*
Pseudomonadota	*Rhodoferax*	K00370	*narG, narZ, nxrA*
Pseudomonadota	*Rhodoferax*	K04561	*norB*
Pseudomonadota	*Rhodoferax*	K02575	*NRT, narK, nrtP, nasA*
Pseudomonadota	*Rhodoferax*	K02305	*norC*
Pseudomonadota	*Rhodoferax*	K00372	*nasA*
Pseudomonadota	*Rhodoferax*	K00376	*nosZ*
Pseudomonadota	*Rhodoferax*	K00368	*nirK*
Pseudomonadota	*Rhodoferax*	K10946	*pmoC-amoC*
Pseudomonadota	*Rhodoferax*	K10535	*hao*
Pseudomonadota	*Rhodoferax*	K02591	*nifK*
Pseudomonadota	*Rhodoferax*	K02586	*nifD*
Pseudomonadota	*Paraburkholderia*	K00371	*narH, narY, nxrB*
Pseudomonadota	*Paraburkholderia*	K00370	*narG, narZ, nxrA*
Pseudomonadota	*Paraburkholderia*	K04561	*norB*
Pseudomonadota	*Pseudomonas*	K02567	*napA*
Pseudomonadota	*Burkholderia*	K00362	*nirB*
Pseudomonadota	*Burkholderia*	K04561	*norB*
Pseudomonadota	*Comamonas*	K00370	*narG, narZ, nxrA*
Pseudomonadota	*Sphingomonas*	K00374	*narI, narV*
Pseudomonadota	*Sphingomonas*	K00371	*narH, narY, nxrB*
Pseudomonadota	*Sphingomonas*	K02575	*NRT, narK, nrtP, nasA*
Pseudomonadota	*Sphingomonas*	K00372	*nasA*
Pseudomonadota	*Hydrogenophaga*	K00371	*narH, narY, nxrB*
Pseudomonadota	*Hydrogenophaga*	K00370	*narG, narZ, nxrA*
Pseudomonadota	*Hydrogenophaga*	K02575	*NRT, narK, nrtP, nasA*
Pseudomonadota	*Hydrogenophaga*	K00372	*nasA*

Pseudomonadota	*Paracoccus*	K00370	*narG, narZ, nxrA*
Pseudomonadota	*Polynucleobacter*	K00374	*narI, narV*
Pseudomonadota	*Polynucleobacter*	K00370	*narG, narZ, nxrA*
Pseudomonadota	*Polynucleobacter*	K02575	*NRT, narK, nrtP, nasA*
Pseudomonadota	*Novosphingobium*	K00374	*narI, narV*
Pseudomonadota	*Novosphingobium*	K02575	*NRT, narK, nrtP, nasA*
Pseudomonadota	*Novosphingobium*	K00372	*nasA*
Pseudomonadota	*Diaphorobacter*	K00370	*narG, narZ, nxrA*
Pseudomonadota	*Mesorhizobium*	K00371	*narH, narY, nxrB*
Pseudomonadota	*Mesorhizobium*	K02575	*NRT, narK, nrtP, nasA*
Pseudomonadota	*Mesorhizobium*	K00372	*nasA*
Bacteroidota	*Flavobacterium*	K00376	*nosZ*
Pseudomonadota	*Magnetospirillum*	K00371	*narH, narY, nxrB*
Pseudomonadota	*Magnetospirillum*	K02575	*NRT, narK, nrtP, nasA*
Pseudomonadota	*Limnohabitans*	K00374	*narI, narV*
Pseudomonadota	*Limnohabitans*	K00370	*narG, narZ, nxrA*
Pseudomonadota	*Limnohabitans*	K02575	*NRT, narK, nrtP, nasA*
